# Photon-counting CT for dynamic lung perfusion: validation of a low-dose protocol in a porcine lung transplantation model

**DOI:** 10.1186/s41747-026-00723-0

**Published:** 2026-04-27

**Authors:** Anna M. Hunkemöller, Thomas Werncke, Julian Dittrich, Cornelia Schaefer-Prokop, Franz Söbbeler, Murat Avsar, Jawad Salman, Arjang Ruhparwar, Rainer Blasczyk, Sevval Besli, Constanca Figueiredo, Alicia Enzig-Strohm, Frank K. Wacker, Hoen-oh Shin

**Affiliations:** 1 Institute of Diagnostic and Interventional Radiology, Hannover Medical School, Carl-Neuberg-Str. 1, 30655 Hannover, Germany; 2 Biomedical Research in Endstage and Obstructive Lung Disease Hannover (BREATH), German Center for Lung Research (DZL), Hannover, Germany; 3 Department of Radiology, Radboud University, Geert Grooteplein Zuid 32, Nijmegen, 6525 GA The Netherlands; 4 Department of Radiology, Meander Medical Center, Maatweg 3, Amersfoort, 3813 TZ The Netherlands; 5 Institute of Transfusion Medicine and Transplant Engineering, Hannover Medical School, Carl-Neuberg-Str. 1, 30655 Hannover, Germany; 6 Department of Cardiothoracic, Transplant and Vascular Surgery, Hannover Medical School, Carl-Neuberg-Str. 1, 30655 Hannover, Germany

**Keywords:** Dynamic lung perfusion, Low-dose CT, Quantitative perfusion imaging, Photon-counting CT, Pulmonary transplantation model

## Abstract

**Objective:**

Photon-counting CT (PCCT) combines improved dose efficiency with spectral imaging, enabling dynamic functional imaging at chest CT dose levels. Dual energy CT typically uses perfused blood volume (PBV) as a static perfusion surrogate. This study compared low-dose dynamic PCCT compared with reference-dose PCCT and static PBV imaging.

**Materials and methods:**

Six minipigs with left lung transplants underwent dynamic perfusion imaging using PCCT at reference and low-dose settings, along with a static PBV scan. Perfusion metrics—Blood Flow Deconvolution (BFD), Mean Transit Time Deconvolution (MTTD), Flow Extraction Product (FEP), and Time to Start Deconvolution (TTSD)—were normalized and analyzed across six lung regions using Kruskal-Wallis tests and Bland-Altman analysis.

**Results:**

Low-dose and reference-dose dynamic PCCT showed strong agreement across perfusion parameters (BVP bias: 0.03; BVD bias: 0.04), with no significant differences in BVP (*p* = 0.995) or BVD (*p* = 0.374). Kinetic metrics were stable across dose levels (all *p* > 0.2). While low-dose imaging showed slightly greater perfusion heterogeneity, BVP remained robust. Static PBV differed significantly from dynamic BVP (reference dose: *p *< 0.001; low-dose: *p* = 0.04). Left-right perfusion differences were detected in two animals by all methods. Estimated doses were 2.37 mSv (reference-dose) and 1.36 mSv (low-dose), comparable to chest CT (1.49 mSv) and below conventional CT perfusion (3–10 mSv).

**Conclusion:**

Dynamic PCCT enables quantitative lung perfusion imaging at radiation doses comparable to standard chest CT. Low-dose dynamic PCCT shows strong agreement with reference-dose acquisitions, while dynamic parameters reveal functional differences not captured by static PBV imaging.

**Relevance statement:**

Dynamic low-dose photon-counting computed tomography enables lung perfusion quantification at radiation doses comparable to standard chest CT, facilitating dose-efficient functional imaging in pulmonary disease.

**Key Points:**

- Low-dose PCCT (~ 1.36 mSv) is feasible, comparable to single chest CT (1.49 mSv).
- Strong agreement was seen between low- and reference-dose PCCT (BVP bias 0.03; BVD bias 0.04).
- Kinetic perfusion metrics remained stable across dose levels (all *p* > 0.2).

**Graphical Abstract:**

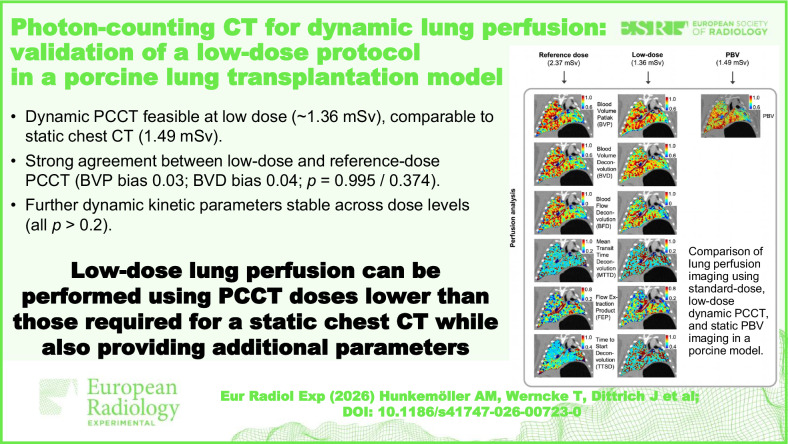

## Introduction

Accurate assessment of regional lung perfusion is critical for diagnosing and managing a wide range of pulmonary disorders, including pulmonary embolism, chronic obstructive pulmonary disease (COPD), chronic thromboembolic pulmonary hypertension and assessment after lung transplantation [1–5]. Detecting perfusion abnormalities or ventilation–perfusion mismatches is essential for disease staging, therapeutic planning, and monitoring treatment response.

Functional imaging techniques such as ventilation/perfusion (V/Q) scintigraphy, MRI, and dynamic contrast-enhanced CT each offer distinct benefits and limitations. While V/Q scintigraphy is sensitive to perfusion defects, its low spatial resolution and reduced diagnostic confidence in structurally abnormal lungs limit diagnostic utility [6]. Perfusion MRI, although radiation-free and capable of providing functional data, remains technically complex and is not universally accessible [7, 8].

Static perfused blood volume (PBV) mapping, especially when implemented with dual-energy CT, has been explored as a surrogate for dynamic CT perfusion [9, 10]. However, PBV imaging captures only a single time point, failing to represent the temporal dynamics of pulmonary perfusion. In contrast, dynamic CT perfusion enables quantitative assessment through kinetic modeling techniques such as Patlak analysis or deconvolution methods. Despite offering high spatial resolution and temporal hemodynamic data, its widespread clinical use has been limited by concerns over radiation dose and image noise, especially in multiphase acquisitions [11, 12]. Efforts to reduce radiation exposure often result in increased image noise, potentially undermining the reliability of perfusion metrics. Furthermore, the lack of standardized quantification and segmentation protocols continues to hinder the broader adoption of dynamic perfusion imaging in clinical practice.

Photon-counting computed tomography (PCCT) introduces a paradigm shift in CT imaging by enabling direct energy discrimination, near-zero electronic noise, enhanced contrast resolution, and improved dose efficiency [13–16]. The technology allows excellent tissue characterization and offers the potential to conduct dynamic functional imaging—such as lung perfusion—at radiation doses comparable to standard morphologic chest CT scans [17]. This integration of anatomical and functional imaging in a single, ultra-low-dose protocol is particularly advantageous for longitudinal monitoring in chronic disease and for research requiring repeated measurements.

This study aims to evaluate dynamic lung perfusion using PCCT at a radiation dose equivalent to standard chest energy-integrating CT. A controlled animal model with defined regional perfusion differences— established via allogeneic left lung transplantation—is used for validation. Results are compared with both reference-dose PCCT and static PBV imaging.

## Materials and methods

### Study design and animal model

Animal experiments were conducted in accordance with the German Animal Welfare Act and the EU Directive 2010/63. All procedures were approved by the Lower Saxony State Office for Consumer Protection and Food Safety (LAVES) under permit number 33.12-42502-04-22-00159. Six female Göttingen minipigs were used as a physiologically relevant model for studying regional perfusion mismatch in PCCT. They underwent orthotopic left allogeneic lung transplantation 69 ± 5 months prior to PCCT, allowing regional perfusion mismatch to be studied. This model allowed for direct, intra-subject comparisons between the native right lung and the transplanted left lung. This facilitated the evaluation of perfusion quantification techniques under controlled and reproducible conditions.

### Animal preparation

Prior to PCCT imaging, pigs underwent a standardized anesthesia and preparation protocol to ensure consistent imaging conditions and physiological stability. Sedation was initiated via intramuscular administration of 5 mg/kg tiletamine/zolazepam, 0.3 mg/kg butorphanol, and 0.04 mg/kg atropine. Following sedation, an intravenous catheter was placed in the auricular vein to facilitate blood sampling and administration of propofol. After induction with propofol, a second intravenous access was established in the contralateral auricular vein, and the animals were endotracheally intubated. Vital parameters were continuously monitored throughout the procedure. During the PCCT scans, general anesthesia was maintained with isoflurane delivered via volume-controlled mechanical ventilation using an air-oxygen mixture (40% O₂), with a tidal volume of ~ 10 mL/kg ideal body weight, respiratory rate of 10 to 12 breaths/min, and a positive end-expiratory pressure of 5 cmH₂O. For scans in inspiration, the pressure-limiting valve was set to 20 cmH₂O, while expiratory scans were performed in apnea at 0 cmH₂O. Upon completion, animals were extubated after showing spontaneous breathing and swallowing reflex under close monitoring.

### Image acquisition

To enable appropriate comparisons, two dynamic PCCT protocols were implemented: a reference protocol using an empirically optimized radiation dose, and a reduced-dose protocol designed to match/fall below the dose of routine clinical chest PCCT examinations [17].

All imaging was performed using a NAEOTOM Alpha PCCT scanner (Siemens Healthineers). Pigs were scanned in the prone position during the inspiratory phase. Each animal underwent three distinct imaging protocols:

1. Dynamic reference-dose PCCT
2. Dynamic low-dose PCCT
3. Static single-phase PBV imaging (low dose)

To minimize residual contrast enhancement between acquisitions, a 5-min delay was introduced between scans. Reference scan parameters were normalized to a 25 cm scan length.

Detailed acquisition parameters for each protocol are summarized in Table 1.

**Table 1:** PCCT acquisition parameters for dynamic perfusion imaging

Parameter	Dynamic reference dose	Dynamic low dose	Static PBV scan
Pig Position	Prone	Prone	Prone
Respiration phase	Inspiration	Inspiration	Inspiration
Scan and display FOV (pixels)	Adapted to body size (512 × 512)	Adapted to body size (512 × 512)	Adapted to body size (512 × 512)
Image quality level	5	3	70
Tube voltage (kV)	90	90	140
Gantry rotation time (s)	0.25	0.25	0.25
Scan range (mm)	254.8	254.8	400
Number of volumes	Variable (19–32)	Variable	1
CTDI per volume (mGy)	0.20 ± 0.04	0.12 ± 0.02	2.4 ± 0.3
DLP, per volume (mGy*cm)	5.4 ± 0.8*	3.1 ± 0.4*	68 ± 5
Pitch	1	1	1.4
Contrast material administration	10 mL Iomeprol 400 + 100 mL Nacl @ 4 mL/s	10 mL Iomeprol 400 + 100 mL Nacl @ 4 mL/s	70 mL Iomeprol +50 mL NaCl
X-ray delay (s)	2	2	-
Bolus tracking	No	No	Aorta ascendens Δ100 HU @120 kV
Cycle time (s)	1.8 (12 * 1.9 = 21.6 s)	1.8 (20 * 1.8 = 36 s)	-
Section thickness (mm)	1	1	1
Section interval (mm)	0.7	0.7	0.7
Convolution kernel	Br40	Br40	Br40
QIR level	3	3	3
**Extrapolated dose values for dynamic perfusion with 20 time points (on a 35 cm scan length)**			
DLP (mGy * cm)	108.0	62.0	68.0
DLP (mGy * cm)	151.2	86.8	95.2
Effective dose (mSv)	2.37	1.36	1.49

*CTDI* CT dose index, *DLP* Dose-length product, *FOV* Field of view, *PBV* Perfused blood volume, *QIR* Quantum iterative reconstruction

*Corresponding to a scan length of 25 cm

### Lung segmentation and analysis

Lung segmentation was performed using custom in-house software developed in MATLAB R2024b (MathWorks), followed by manual correction in ITK Snap (4.2.2) by a single primary observer (A.M.H.). All segmentations were reviewed and supervised by a second experienced thoracic radiologist (H.O.S.). In cases of uncertainty, consensus was reached through joint review. Each lung was anatomically divided into apical, middle, and basal regions of approximately equal volume. To enable direct regional comparison, perfusion parameters were normalized to a [0, 1] scale within each individual animal.

### Perfusion analysis

**Fig. 1:**
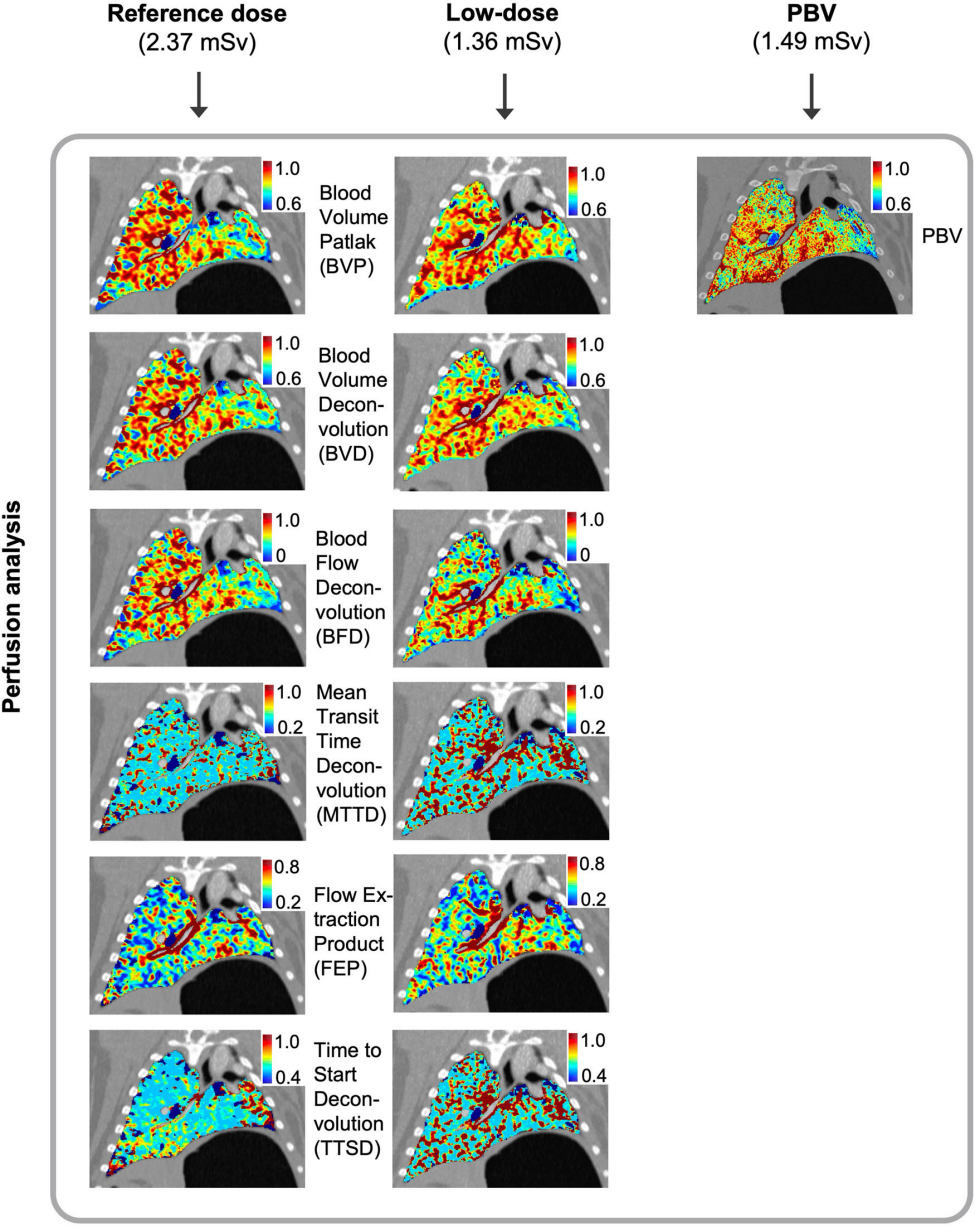
Experimental workflow for lung perfusion imaging using photon-counting CT (PCCT) in a porcine transplantation model. Six Göttingen minipigs underwent allogeneic left lung transplantation, followed by PCCT imaging using three protocols: dynamic perfusion at reference dose (left column), dynamic perfusion at reduced dose (middle column), and static perfused blood volume (PBV) imaging (right column). Dynamic perfusion maps were reconstructed using Patlak modeling (blood volume Patlak, BVP) and deconvolution-based methods, including blood volume (BVD), blood flow (BFD), mean transit time (MTTD), flow extraction product (FEP), and time to start (TTSD). Color-coded parametric maps depict quantitative perfusion across lung regions for each protocol

Dynamic perfusion data were analyzed using both Patlak and deconvolution-based kinetic modeling to extract BVP, BVD, BFD, MTTD, FEP, and TTSD. Static PBV was derived from iodine maps obtained in static dual-energy CT images (Fig. 1). Image analysis and post-processing were performed using syngo.via (Siemens Healthineers)

### Statistical methods

All statistical evaluations were performed using JMP 18.0.2 (JMP Statistical Discovery LLC). Regional differences of perfusion parameters in all three scan protocols were evaluated using non-parametric Kruskal-Wallis tests; Bland-Altman plots were used to assess agreement. Statistical significance was set at *p* < 0.05. Agreement between dynamic and static metrics was further assessed using Bland-Altman analysis (mean bias ± 1.96 SD as limits of agreement). Results are reported as mean ± standard deviation, or median (interquartile range) as appropriate.

## Results

### Animal model

All six animals (see Table 2) completed the imaging protocol without adverse events.

**Table 2:** Characteristics of the animal model

Parameter	Value
Total number of pigs	6
Breed	Göttinger Minipig
Sex (% female)	100
Age at lung transplantation (months)	13.5 ± 0.6
Time since transplantation (months)	69.0 ± 4.9
Age at time of scan (months)	82.4 ± 5.0
Weight at CT scan (kg)	46.5 ± 3.8

Data are presented as mean ± standard deviation where applicable

### Dynamic perfusion quantification

**Fig. 2:**
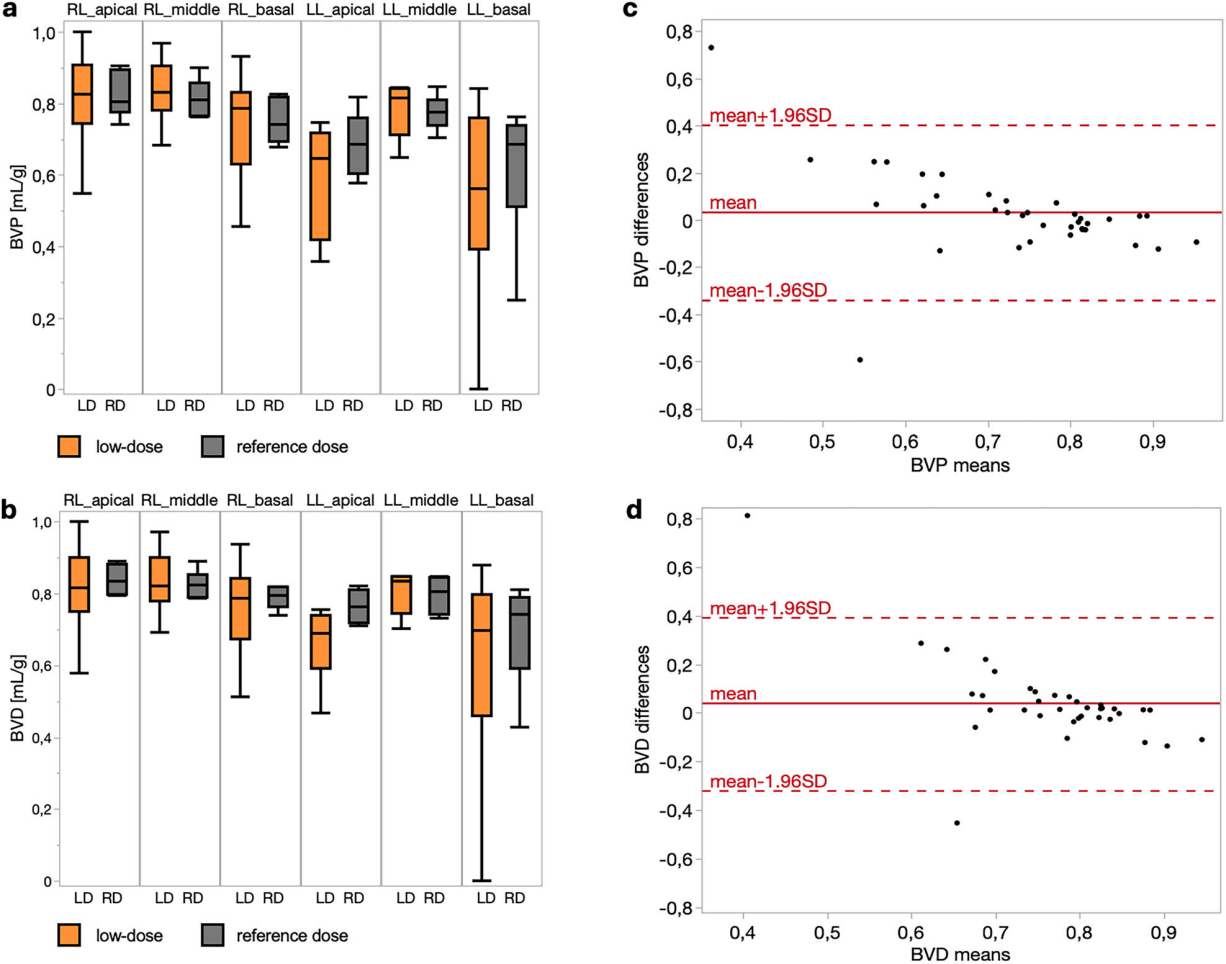
Regional comparison of Blood Volume Patlak (BVP) and Blood Volume Deconvolution (BVD) between dynamic reference dose (RD) and low-dose (LD) photon-counting CT (PCCT). Comparison of blood volume parameters between reference dose and low-dose CT scans. **a** Boxplots of BVP across different lung regions for reference dose (gray) and low-dose (orange) acquisitions. **b** Boxplots of BVD showing regional distributions for both dose levels. **c** Bland–Altman plot illustrating the agreement between reference dose and low-dose measurements for BVP, with red lines representing the mean difference and ±1.96 standard deviations (SD). **d** Bland–Altman plot for BVD, indicating the consistency between dose levels

**Fig. 3:**
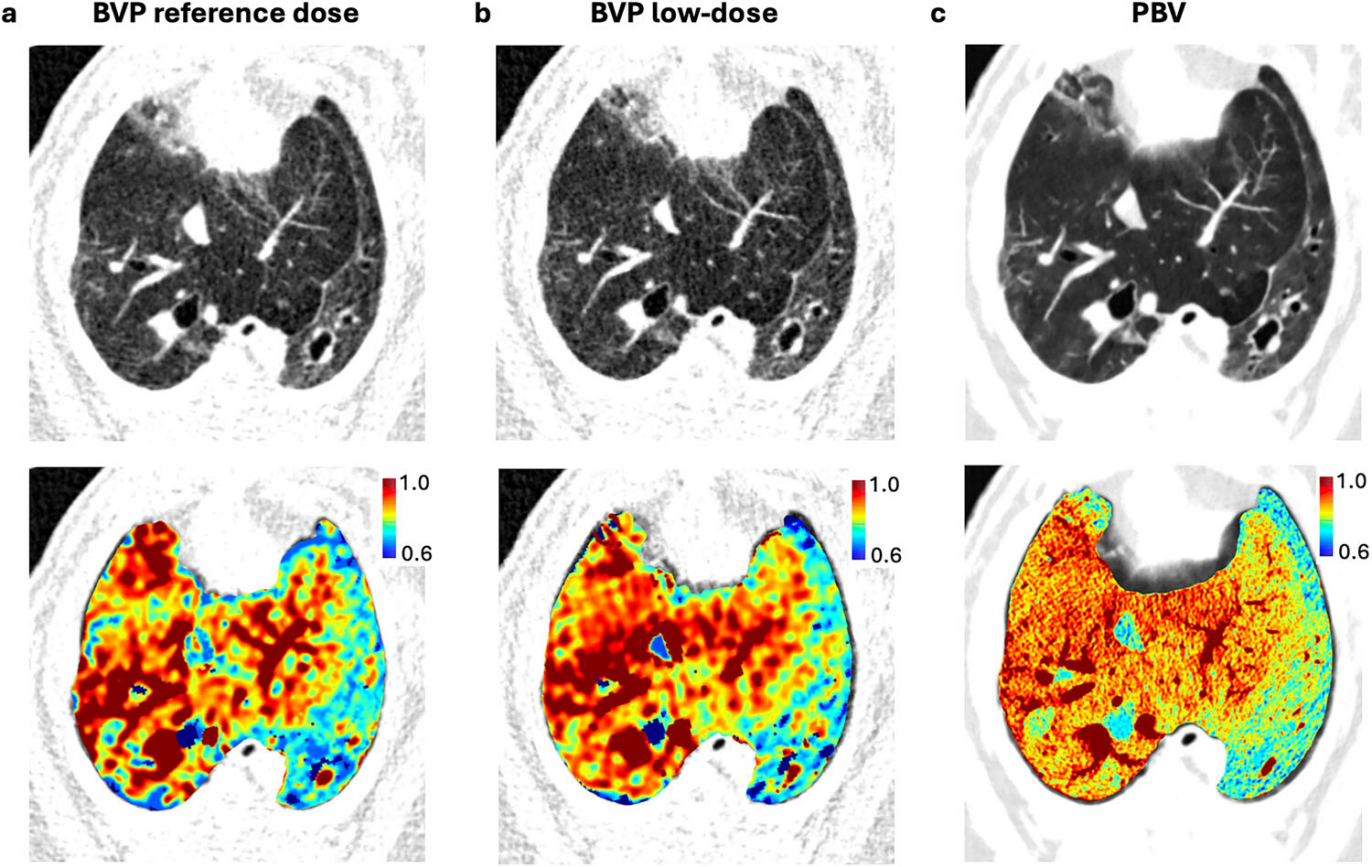
Comparison of lung perfusion imaging using standard-dose, low-dose dynamic PCCT, and static PBV imaging in a porcine model. Axial attenuation images (top row) and corresponding perfusion maps (bottom row) in Pig 1 acquired using dynamic photon-counting CT (PCCT) with blood volume Patlak (BVP) at standard dose (**a**), low dose (**b**), and static perfused blood volume (PBV) imaging (**c**). Relative perfusion is color-coded from 0.6 (blue) to 1.0 (red) (normalized to [0, 1]). All display windows were standardized to mean ± standard deviation

Strong agreement was observed between dynamic low-dose and reference-dose PCCT across all perfusion metrics. Boxplots demonstrate comparable regional distributions of both BVP and BVD across all six lung segments with both protocols (Fig. 2a, b). Despite similar mean values, standard deviations were significantly increased in the low-dose group for both BVP (*p* = 0.011) and BVD (*p* = 0.001), suggesting greater measurement variability under dose-reduced conditions. PBV imaging exhibits higher spatial resolution but less detailed perfusion heterogeneity and differs quantitatively from dynamic blood volume measurements (Fig. 3).

Distributional symmetry, as assessed by skewness, was preserved across protocols (BVP: *p* = 0.875; BVD: *p* = 0.901). Kurtosis remained stable for BVP (*p* = 0.184) but was significantly elevated for BVD in the low-dose setting (*p* = 0.001), highlighting the increased susceptibility of deconvolution-based BVD to image noise. Collectively, these results suggest that Patlak-derived BVP provides superior stability and noise resilience compared to BVD in low-dose dynamic imaging.

**Fig. 4:**
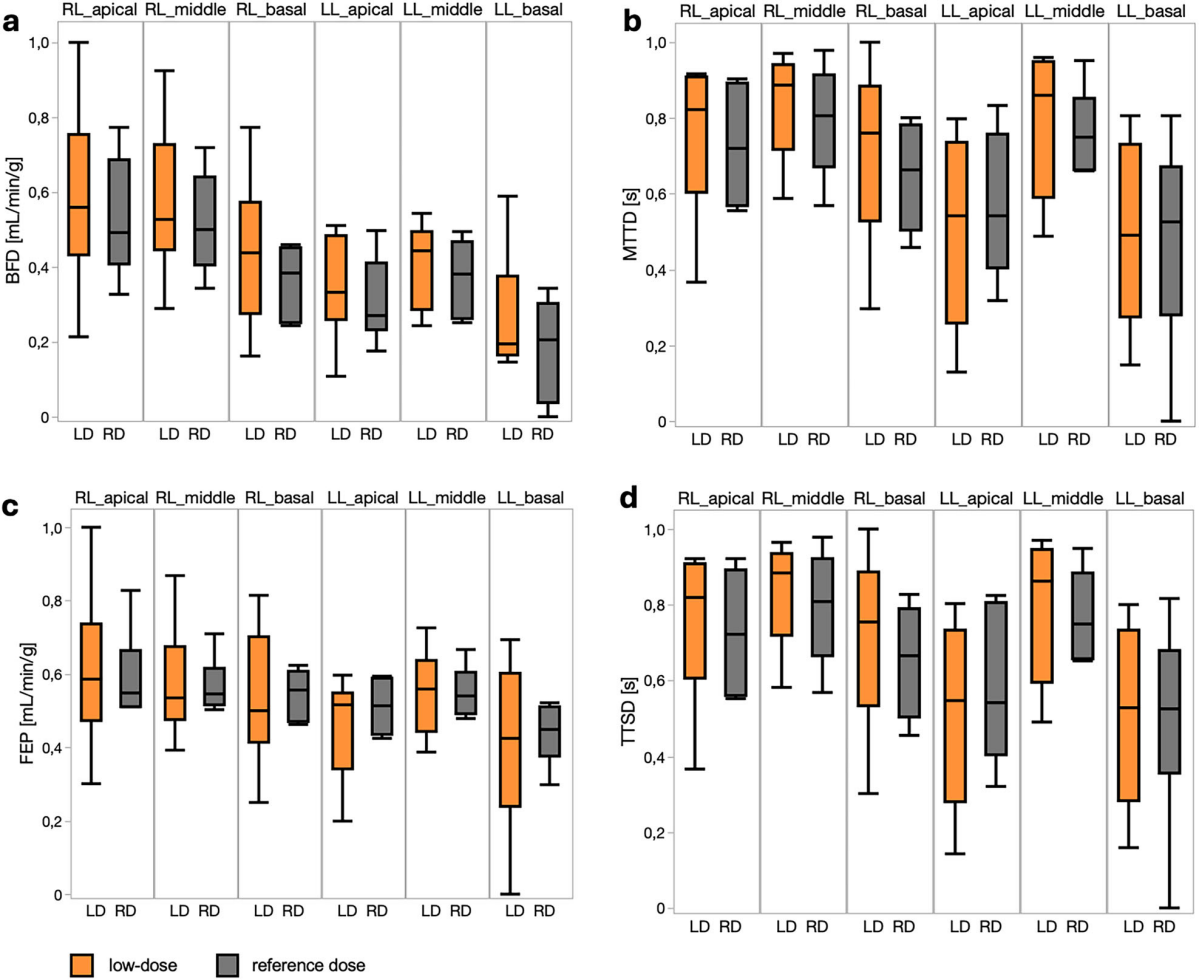
Regional comparison of additional dynamic lung perfusion parameters derived from Blood Flow Deconvolution (BFD), Mean Transit Time Deconvolution (MTTD), Flow Extraction Product (FEP), and Time To Start Deconvolution (TTSD) between low-dose (LD, orange) and reference-dose (RD, gray) PCCT scans. Boxplots display median, interquartile range, and full data range for six anatomically defined lung regions–right lung (RL) apical, middle, basal; left lung (LL) apical, middle, basal. **a** BFD reflects blood flow normalized to tissue mass (mL/min/g) and shows comparable distributions between LD and RD across all regions, with slightly increased variability in LD basal regions. **b** MTTD indicates average contrast transit time (seconds) through the lung microvasculature, exhibiting consistent median values and overlapping ranges between dose levels, underscoring stable temporal dynamics. **c** FEP, representing combined flow and extraction capacity (mL/min/g), demonstrates similar medians and spreads across LD and RD conditions, supporting preserved functional measurement at reduced dose. **d** TTSD, calculated with the deconvolution model to represent the start time of tissue enhancement, shows overlapping distributions between LD and RD conditions, indicating comparable timing of perfusion onset

Other dynamic perfusion metrics—including BFD, MTTD, FEP, and TTSD—were not significantly affected by dose level (all *p* > 0.2; Table 3 and Fig. 4). Together, these findings illustrate minimal dose-dependent bias or variability in advanced perfusion kinetics, confirming that low-dose PCCT protocols maintain quantitative accuracy and temporal resolution necessary for comprehensive lung perfusion assessment.

**Table 3:** Additional regional perfusion parameters across all pigs

	Dynamic reference dose	Dynamic low-dose	*p*-value
Blood flow deconvolution (BFD) (mL/min/g)			
LL apical	0.27 (0.23–0.41)	0.33 (0.26–0.49)	0.233
LL middle	0.38 (0.26–0.47)	0.44 (0.29–0.49)	
LL basal	0.21 (0.04–0.30)	0.19 (0.17–0.38)	
RL apical	0.49 (0.41–0.69)	0.56 (0.43–0.76)	
RL middle	0.5 (0.41–0.64)	0.53 (0.45–0.73)	
RL basal	0.38 (0.25–0.45)	0.44 (0.28–0.57)	
Mean transit time deconvolution (MTTD) (s)			
LL apical	0.54 (0.40–0.76)	0.54 (0.26–0.74)	0.434
LL middle	0.75 (0.66–0.85)	0.86 (0.59–0.95)	
LL basal	0.52 (0.28–0.67)	0.49 (0.28–0.73)	
RL apical	0.72 (0.57–0.89)	0.82 (0.60–0.91)	
RL middle	0.81 (0.67–0.91)	0.89 (0.72–0.94)	
RL basal	0.66 (0.51–0.78)	0.76 (0.53–0.88)	
Flow extraction product (FEP) (mL/min/g)			
LL apical	0.51 (0.44–0.59)	0.52 (0.34–0.55)	0.915
LL middle	0.54 (0.49–0.60)	0.56 (0.44–0.64)	
LL basal	0.45 (0.38–0.51)	0.42 (0.24–0.60)	
RL apical	0.55 (0.51–0.66)	0.59 (0.47–0.74)	
RL middle	0.54 (0.52–0.62)	0.54 (0.48–0.67)	
RL basal	0.56 (0.47–0.61)	0.50 (0.41–0.70)	
Time to Start Deconvolution (TTSD) (s)			
LL apical	0.54 (0.40–0.80)	0.55 (0.28–0.73)	0.482
LL middle	0.75 (0.66–0.88)	0.86 (0.59–0.95)	
LL basal	0.53 (0.36–0.68)	0.53 (0.28–0.73)	
RL apical	0.72 (0.56–0.89)	0.82 (0.61–0.91)	
RL middle	0.81 (0.66–0.92)	0.88 (0.72–0.94)	
RL basal	0.67 (0.50–0.79)	0.76 (0.53–0.89)	

Values are presented as median (interquartile range). *p*-values correspond to Kruskal-Wallis tests comparing reference-dose and low-dose acquisition across all six lung regions

*LL* Left lung lobe, *RL* Right lung lobe

Bland-Altman analysis demonstrated minimal bias and narrow limits of agreement for BVP (mean bias: 0.03; limits of agreement: -0.34 to 0.40) and BVD (mean bias: 0.04; limits: -0.32 to 0.40), indicating robust concordance between protocols (Table 4 and Fig. 2c, d). No significant differences were detected in BVP (*p* = 0.995) or BVD (*p* = 0.374) between dose levels.

**Table 4:** Regional perfusion analysis across all pigs

Parameter	Acquisition		
	Dynamic reference dose	Dynamic low-dose	*p*-value
Blood volume Patlak (BVP) (mL/g)			
LL apical	0.69 (0.60–0.76)	0.65 (0.42–0.72)	0.955
LL middle	0.77 (0.74–0.81)	0.82 (0.71–0.84)	
LL basal	0.68 (0.51–0.74)	0.56 (0.39–0.76)	
RL apical	0.80 (0.77–0.90)	0.83 (0.74–0.91)	
RL middle	0.81 (0.77–0.86)	0.83 (0.78–0.91)	
RL basal	0.74 (0.69–0.82)	0.79 (0.63–0.83)	
BVP SD (mL/g)			
LL apical	0.45 (0.38–0.57)	0.47 (0.33–0.76)	0.011
LL middle	0.09 (0.05–0.20)	0.13 (0.06–0.29)	
LL basal	0.22 (0.09–0.41)	0.45 (0.15–0.65)	
RL apical	0.12 (0.02–0.20)	0.18 (0.08–0.31)	
RL middle	0.12 (0.07–0.16)	0.12 (0.10–0.23)	
RL basal	0.13 (0.05–0.18)	0.19 (0.16–0.41)	
BVP kurtosis			
LL apical	0.15 (0.08–0.22)	0.08 (0.02–0.14)	0.184
LL middle	0.27 (0.20–0.49)	0.34 (0.15–0.49)	
LL basal	0.16 (0.10–0.27)	0.06 (0.04–0.29)	
RL apical	0.38 (0.18–0.72)	0.29 (0.19–0.44)	
RL middle	0.31 (0.19–0.48)	0.34 (0.22–0.42)	
RL basal	0.26 (0.15–0.45)	0.24 (0.10–0.30)	
BVP skewness			
LL apical	0.36 (0.25–0.42)	0.41 (0.32–0.54)	0.875
LL middle	0.26 (0.06–0.34)	0.17 (0.06–0.32)	
LL basal	0.33 (0.21–0.41)	0.43 (0.18–0.50)	
RL apical	0.36 (0.13–0.63)	0.25 (0.13–0.50)	
RL middle	0.26 (0.12–0.41)	0.17 (0.14–0.32)	
RL basal	0.28 (0.13–0.47)	0.23 (0.19–0.38)	
Blood volume deconvolution (BVD) (mL/g)			
LL apical	0.76 (0.72–0.81)	0.69 (0.59–0.74)	0.374
LL middle	0.81 (0.74–0.84)	0.84 (0.74–0.85)	
LL basal	0.74 (0.59–0.79)	0.70 (0.46–0.80)	
RL apical	0.83 (0.80–0.88)	0.82 (0.75–0.90)	
RL middle	0.82 (0.79–0.85)	0.82 (0.78–0.90)	
RL basal	0.80 (0.76–0.82)	0.79 (0.67–0.84)	
BVD SD (mL/g)			
LL apical	0.16 (0.08–0.21)	0.46 (0.34–0.62)	0.001
LL middle	0.06 (0.04–0.13)	0.11 (0.10–0.23)	
LL basal	0.06 (0.01–0.30)	0.22 (0.09–0.56)	
RL apical	0.06 (0.03–0.09)	0.16 (0.11–0.33)	
RL middle	0.14 (0.11–0.16)	0.15 (0.14–0.27)	
RL basal	0.07 (0.04–0.10)	0.22 (0.18–0.34)	
BVD kurtosis			
LL apical	0.37 (0.30–0.53)	0.10 (0.05–0.17)	0.001
LL middle	0.57 (0.38–0.73)	0.50 (0.27–0.54)	
LL basal	0.57 (0.30–0.71)	0.31 (0.08–0.54)	
RL apical	0.55 (0.49–0.70)	0.36 (0.20–0.49)	
RL middle	0.42 (0.38–0.49)	0.40 (0.23–0.42)	
RL basal	0.61 (0.47–0.65)	0.29 (0.19–0.33)	
BVD skewness			
LL apical	0.28 (0.26–0.46)	0.44 (0.37–0.51)	0.901
LL middle	0.28 (0.19–0.47)	0.26 (0.18–0.33)	
LL basal	0.31 (0.12–0.45)	0.33 (0.17–0.47)	
RL apical	0.58 (0.25–0.79)	0.30 (0.24–0.50)	
RL middle	0.36 (0.26–0.55)	0.30 (0.26–0.40)	
RL basal	0.31 (0.18–0.49)	0.32 (0.29–0.38)	

Values are presented as median (interquartile range). *p*-values correspond to Kruskal–Wallis tests comparing reference-dose and low-dose acquisition across all six lung regions

*LL* Left lung lobe, *RL* Right lung lobe

### Detection of regional perfusion abnormalities

**Fig. 5:**
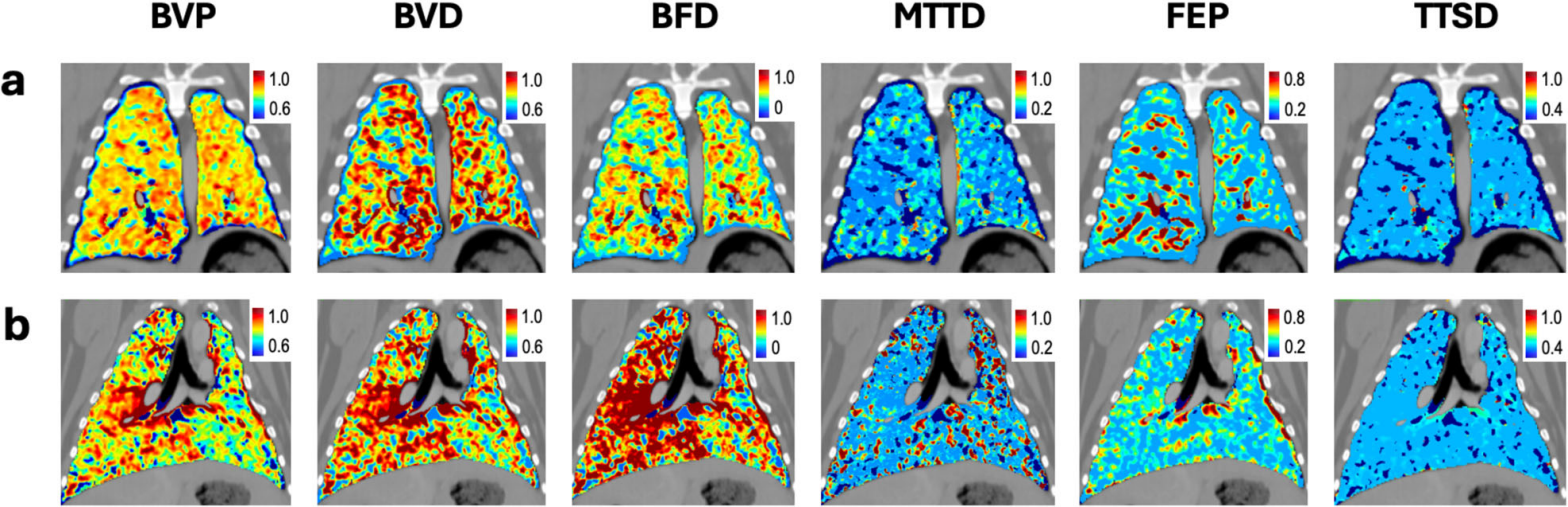
Representative perfusion maps derived from volumetric analysis in the coronary view. **a** Animal without detectable left–right volumetric perfusion differences, demonstrating homogeneous distribution across all functional parameters, including blood volume perfusion (BVP), blood volume density (BVD), blood flow density (BFD), mean transit time (MTTD), flow extraction product (FEP), and time-to-start (TTSD). **b** Animal with asymmetric (left–right) perfusion differences, most pronounced in BVP, BVD, and BFD maps. Parametric maps are displayed as relative perfusion values with color coding normalized to the range [0–1]. In **b**, the right lung shows higher perfusion values compared to **a**, indicating compensatory hyperperfusion

In two of six pigs, both dynamic and static PCCT protocols consistently identified regional perfusion differences between the transplanted (left) and native (right) lungs. These two animals demonstrated pronounced left–right asymmetry across both blood volume-based and functional parameters, indicating a perfusion impairment in the transplanted lung. In the remaining four animals, no left-right differences were observed by either method. Representative examples of animals with and without perfusion asymmetry are shown in Fig. 5.

Interestingly, the functional perfusion parameters BFD, FEP, MTTD, and TTSD consistently differed between the left and right lungs, also in the remaining four animals with visually symmetric perfusion. In both the reference-dose and low-dose protocols, the left lung demonstrated lower BFD, lower FEP, shorter TTSD, and reduced MTTD compared with the right lung. In the reference-dose setting, these differences reached statistical significance for BFD (*p* = 0.04) and FEP (*p* = 0.01), while the remaining parameters did not reach statistical significance but displayed consistent trends in the same direction. Importantly, these inter-lung differences were only detectable numerically but did not translate into conspicuous visual differences on standardized color-coded perfusion maps.

### Static PBV *versus* dynamic perfusion metrics

PBV values remained consistent between dose protocols; however, they significantly diverged from dynamic BVP measurements (reference dose: *p* < 0.001; low dose: *p* = 0.04).

### Radiation dose efficiency

The effective dose for a single dynamic PCCT acquisition was 0.0085 mSv using the reference protocol (DLP = 5.4 mGy·cm), and 0.0049 mSv for the low-dose protocol (DLP = 3.1 mGy·cm), calculated using a standard chest CT conversion factor of 0.0157 mSv·mGy⁻¹·cm⁻¹. All values were normalized to a scan length of 25 cm, which is slightly below the typical thoracic coverage in clinical chest CT (commonly 30–35 cm). Extrapolated to 35 cm scan length, the cumulative DLP for 20 dynamic acquisitions reached 151.2 mGy·cm for the reference protocol and 86.8 mGy·cm for the low-dose protocol, corresponding to effective doses of 2.37 mSv and 1.36 mSv, respectively, with a time resolution of 1.8 s (Table 1). For comparison, a single static PBV scan yielded an extrapolated DLP of 95.2 mGy·cm and an effective dose of 1.49 mSv.

## Discussion

This study shows that dynamic low-dose PCCT enables accurate, reproducible quantification of lung perfusion in a porcine allogeneic transplantation model. Quantitative parameters derived from the low-dose protocol showed good concordance with those from a dose-optimized reference dynamic PCCT, confirming the robustness of the technique. Notably, radiation exposure was comparable to or lower than routine chest PCCT, significantly reducing the burden typically associated with perfusion imaging. Key perfusion indices—including BVP, BVD, BFD, MTTD and TTSD—remained quantitatively stable under low-dose conditions, confirming the feasibility of dynamic PCCT at markedly reduced radiation exposure. The Patlak-derived BVP was more robust, exhibiting superior noise resistance compared to deconvolution-based BVD, underscoring its technical reliability for quantitative lung perfusion assessment.

In two animals, both BVP/BVD and PBV detected perfusion deficits in transplanted lobes, demonstrating the clinical sensitivity and reproducibility of the technique for identifying physiologically relevant regional abnormalities. However, in the remaining four animals, BVP/BVD failed to detect any left–right differences, whereas further functional dynamic PCCT parameters clearly differed between the two lungs: the left, transplanted lung consistently exhibited lower BFD and FEP, as well as shorter MTTD and TTSD, in both reference- and low-dose protocols. Notably, these differences were subtle and became evident only through region-based quantitative analysis; they were not readily appreciable on qualitative visual analysis of the color-coded parametric maps. The observed inter-animal variability reflects biological heterogeneity within the long-term transplantation model rather than methodological inconsistency. Importantly, dynamic kinetic parameters remained sensitive to subtle functional asymmetries, underscoring the complementary value of multiparametric dynamic perfusion imaging.

Since BFD, FEP, MTTD, and TTSD can only be derived from dynamic acquisitions, static PBV mapping lacks the temporal resolution to capture contrast kinetics and microvascular transit characteristics. Consequently, dynamic PCCT can reveal subtle functional asymmetries that may precede overt PBV changes, offering a potential advantage for early detection of regional perfusion compromise. The reproducibility of these results across dose levels further supports the robustness of dynamic parameters as complementary biomarkers to static PBV in comprehensive pulmonary perfusion assessment. However, the clinical relevance and prognostic significance of these quantitatively detected yet visually inapparent asymmetries remain to be established. It is conceivable that such parameters reflect early microvascular remodeling or subtle alterations in regional perfusion efficiency, but further validation in larger and longitudinal cohorts is required.

Our findings affirm that the intrinsic advantages of PCCT, such as high spatial resolution, spectral discrimination, and dose efficiency, are beneficial for dynamic perfusion imaging, especially at low doses [18, 19]. Choice of kinetic modeling played a critical role in maintaining quantification accuracy. The Patlak model, based on linear unidirectional tracer kinetics, exhibited greater resistance to low-dose noise and better preservation of statistical descriptors (*e.g*., kurtosis, skewness) than deconvolution-based approaches [20]. BVD, requiring numerical deconvolution, showed higher variability and noise sensitivity at low doses. These findings align with previous simulation and clinical studies advocating dose-specific model selection [21]. While static PBV maps remain clinically useful, they offer only semi-quantitative perfusion estimates. Our findings confirm significant divergence between static PBV and dynamic BVP, particularly in pathological conditions, underscoring the superior diagnostic performance of time-resolved approaches for functional pathology. This discrepancy suggests that while PBV may be useful for visualizing relative regional perfusion deficits, it should not be considered a quantitative surrogate for dynamic BVP in functional imaging assessments [22, 23].

Even with 20 repeated dynamic low-dose scans, the total effective dose remained comparable to or lower than that of a single static perfusion scan, and substantially lower than typical perfusion energy-integrating CT protocols, which range from 3 to 10 mSv. When extrapolated to human-equivalent scan lengths (35 cm), the total effective dose of a dynamic PCCT scan with 20 time points was estimated to be less than 1.4 mSv—comparable to a single PBV scan (~ 1.5 mSv) and still significantly lower than conventional perfusion CT (3–10 mSv).

Dynamic low-dose PCCT addresses longstanding concerns about radiation exposure in perfusion imaging, enabling quantitative, time-resolved pulmonary functional imaging at routine dose levels of standard chest PCCT. The ability to characterize regional perfusion dynamics offers significant potential for guiding personalized treatment strategies in complex pulmonary diseases [17, 19, 24].

### Limitations and future directions

This study has several limitations. The small sample size (*n* = 6) limits statistical power, although consistency across animals and reproducibility of perfusion metrics at 6 lung areas partially mitigate this concern. Inter-species anatomical differences may affect generalizability to human studies, particularly regarding lobar anatomy and perfusion heterogeneity.

Technically, deconvolution-based metrics exhibited increased kurtosis and variability at low dose, reinforcing the need for dose-optimized modeling strategies. Computational complexity remains a practical hurdle, particularly for FEP and TTSD, though automation and AI-based post-processing may address this in the near term [25].

A further limitation of our study is that reproducibility across repeated scans at the respective dose levels was not formally assessed, as each animal underwent just a single dynamic PCCT acquisition per dose level. As such, intra- or inter-scan variability could not be quantitatively evaluated. However, the high concordance between reference-dose and low-dose perfusion parameters, across multiple animals and metrics, provides indirect evidence supporting the robustness and potential reproducibility of the low-dose protocol. Future studies should include repeated measurements under identical conditions to formally establish reproducibility and test–retest reliability. Lastly, although standardized anesthesia and monitoring minimized confounding variables, motion artifacts inherent to animal studies could have modestly affected image quality.

## Data Availability

Data and materials are available on reasonable request.

## Abbreviations

BFD: Blood flow deconvolution
BVD: Blood volume deconvolution
BVP: Blood volume Patlak
DLP: Dose length product
FEP: Flow extraction product
MTTD: Mean transit time deconvolution
PBV: Perfused blood volume
PCCT: Photon-counting CT
TTSD: Time to start deconvolution
